# Roots Mediate the Effects of Snowpack Decline on Soil Bacteria, Fungi, and Nitrogen Cycling in a Northern Hardwood Forest

**DOI:** 10.3389/fmicb.2019.00926

**Published:** 2019-04-30

**Authors:** Patrick O. Sorensen, Jennifer M. Bhatnagar, Lynn Christenson, Jorge Duran, Timothy Fahey, Melany C. Fisk, Adrien C. Finzi, Peter M. Groffman, Jennifer L. Morse, Pamela H. Templer

**Affiliations:** ^1^Lawrence Berkeley National Laboratory, Climate and Ecosystem Sciences Division, Berkeley, CA, United States; ^2^Department of Biology, Boston University, Boston, MA, United States; ^3^Biology Department, Vassar College, Poughkeepsie, NY, United States; ^4^Centre for Functional Ecology, University of Coimbra, Coimbra, Portugal; ^5^Department of Natural Resources, Cornell University, Ithaca, NY, United States; ^6^Department of Biology, Miami University, Oxford, OH, United States; ^7^City University of New York Advanced Science Research Center at the Graduate Center, New York, NY, United States; ^8^Cary Institute of Ecosystem Studies, Millbrook, NY, United States; ^9^Department of Environmental Science and Management, Portland State University, Portland, OR, United States

**Keywords:** elevation gradient, plant roots, soil N cycle, snowpack, soil bacteria and fungi

## Abstract

Rising winter air temperature will reduce snow depth and duration over the next century in northern hardwood forests. Reductions in snow depth may affect soil bacteria and fungi directly, but also affect soil microbes indirectly through effects of snowpack loss on plant roots. We incubated root exclusion and root ingrowth cores across a winter climate-elevation gradient in a northern hardwood forest for 29 months to identify direct (i.e., winter snow-mediated) and indirect (i.e., root-mediated) effects of winter snowpack decline on soil bacterial and fungal communities, as well as on potential nitrification and net N mineralization rates. Both winter snowpack decline and root exclusion increased bacterial richness and phylogenetic diversity. Variation in bacterial community composition was best explained by differences in winter snow depth or soil frost across elevation. Root ingrowth had a positive effect on the relative abundance of several bacterial taxonomic orders (e.g., Acidobacterales and Actinomycetales). Nominally saprotrophic (e.g., Saccharomycetales and Mucorales) or mycorrhizal (e.g., Helotiales, Russalales, Thelephorales) fungal taxonomic orders were also affected by both root ingrowth and snow depth variation. However, when grouped together, the relative abundance of saprotrophic fungi, arbuscular mycorrhizal fungi, and ectomycorrhizal fungi were not affected by root ingrowth or snow depth, suggesting that traits in addition to trophic mode will mediate fungal community responses to snowpack decline in northern hardwood forests. Potential soil nitrification rates were positively related to ammonia-oxidizing bacteria and archaea abundance (e.g., Nitrospirales, Nitrosomondales, Nitrosphaerales). Rates of N mineralization were positively and negatively correlated with ectomycorrhizal and saprotrophic fungi, respectively, and these relationships were mediated by root exclusion. The results from this study suggest that a declining winter snowpack and its effect on plant roots each have direct effects on the diversity and abundance of soil bacteria and fungal communities that interact to determine rates of soil N cycling in northern hardwood forests.

## Introduction

Snowpack depth and duration are critical factors that determine winter soil temperature in mid-latitude temperate ecosystems (Zhang, 2005; Burakowski et al., 2008). The timing of snowpack onset, occurrence of episodic snow events, and total annual snowfall interact with air temperature to determine soil temperature during winter (Isard and Schaetzl, 1998; Henry, 2008; Kreyling and Henry, 2011). Climate models project that by the year 2100 rising air temperatures will reduce the depth of the winter snowpack by approximately 0.5 m and reduce the duration of snow cover by 10 to 20 days compared to a 1979–2008 baseline in the northeastern United States, as well as result in lower winter soil temperatures and an increased number of soil freeze-thaw events (Hayhoe et al., 2007; Campbell et al., 2010; Brown and DeGaetano, 2011). Soil bacteria and fungi are likely to play a pivotal role in the response of temperate forest ecosystems to reductions in the winter snowpack because these soil microbial communities are the principal decomposers of soil organic matter (SOM) and are known to be sensitive to changes in soil temperature (Frey et al., 2013; Pold et al., 2015). However, the relationship between loss of winter snow cover, the taxonomic distribution of soil bacteria and fungi, and potential connections to ecosystem functioning (e.g., soil nitrogen (N) cycling) are not well characterized.

Specific microbial functional groups strongly influence soil N cycling and the effect of winter snowpack loss on their abundance and distribution may be a significant constraint on forest ecosystem function (Isobe and Ohte, 2014). For example, if snowpack decline and increased winter soil freezing cause large reductions in the abundance of ammonia-oxidizing bacteria or archaea, this could have significant impacts on soil nitrification due to limited functional redundancy of this N cycling trait among bacterial lineages (Isobe and Ohte, 2014; Kuypers et al., 2018). Similarly, fungal functional groups that differ in carbon (C) acquisition strategy (e.g., saprotrophic fungi versus ecto-or arbuscular mycorrhizal fungi) produce different exoenzymes with distinct biochemical characteristics and substrate affinities that affect soil N mobilization (Hodge et al., 2001; Read and Perez-Moreno, 2003; Schneider et al., 2012). Thus, changes in the activity or abundance of different fungal functional groups has been shown to alter SOM or litter decomposition, nutrient turnover, and plant N assimilation (Gadgil and Gadgil, 1971; Hobbie and Högberg, 2012; Bodeker et al., 2014; Talbot et al., 2015).

Understanding the effect of declining winter snow depth on root-microbial interactions is also critical because the effect of snowpack reduction on plant roots is likely to lead to changes in microbial community composition and functioning (Sorensen et al., 2016). Winter soil freezing is known to increase overwinter root mortality, damage roots, and decrease root nutrient uptake in the growing season (Comerford et al., 2013; Campbell et al., 2014; Sanders-DeMott et al., 2018; Reinmann and Templer, 2018). Root C exudates can increase the abundance and richness of both fungal and bacterial communities (De Graaff et al., 2010; Shi et al., 2011), so it is possible that reductions in C availability caused by soil freezing-induced mortality of roots will result in reductions in microbial diversity. On the other hand, root production is known to increase in growing seasons following winters with a smaller snowpack compared to ambient conditions (Tierney et al., 2001; Cleavitt et al., 2008; Sorensen et al., 2016) and this compensatory root growth may increase plant-microbe competition for nutrients or water. Hence, the response of soil microbial communities to snowpack decline is likely to be mediated by an interaction between snow-cover loss and its attending effect on plant roots via soil frost damage.

At the Hubbard Brook Experimental Forest (New Hampshire, United States), low elevation sites experience shallower winter snow cover, colder winter soil temperatures, and greater soil frost depth and duration compared to high elevation sites (Groffman et al., 2009). Soil N cycle process rates including N mineralization, nitrification, and denitrification have been found to increase with increased winter snow depth and duration along this elevation gradient (Durán et al., 2014; Morse et al., 2015; Sorensen et al., 2016). In addition, root biomass production has been observed to increase with greater soil frost at lower elevation sites (Sorensen et al., 2016). Here we ask if the observed relationships between soil N cycle process rates and winter snowpack depth and duration are associated with variation in bacterial and fungal communities? If so, do effects of snowpack loss on microbial communities interact with plant roots to mediate soil N cycle rates?

To address these questions, root ingrowth and root exclusion soil cores were incubated *in situ* for 29 months across an elevation gradient to determine the direct (e.g., winter snow- and soil frost-mediated) and indirect (e.g., root-mediated) effects of winter snowpack on soil bacterial and fungal communities, as well as on potential N mineralization and nitrification rates. Bacterial community diversity was hypothesized to be positively related to winter snow depth and duration. Bacterial and fungal community composition were also expected to co-vary with snow depth across the elevation gradient. Root exclusion was hypothesized to result in enrichment of saprotrophic fungi and reductions in mycorrhizal fungi and these trends were expected to increase in magnitude with declining winter snow depth. Lastly, we expected that soil N mineralization rates would be positively associated with saprotrophic fungi and that soil nitrification rates would be positively related to the abundance of ammonia-oxidizing bacteria and archaea.

## Materials and Methods

### Field Site Description

The Hubbard Brook Experimental Forest is located in the White Mountain National Forest in central New Hampshire, United States (43.56°N, 71.45°W). Elevation ranges from approximately 225 m to 1100 m. Maximum annual air temperature is 19°C, minimum annual air temperature is -9°C, and the mean annual temperature has increased by approximately 0.3°C per decade over the last 50 years (Hamburg et al., 2013). Annual precipitation is about 1200 mm, one-third of which occurs as snow (Bailey et al., 2003). Winter snowpack depth is 70-100 cm and winter soil frost depth ranges from 0 to 25 cm depth below the soil surface (Campbell et al., 2010). Over the last 50 years, winter snowpack depth has declined by 26 cm and the duration of winter snow cover has declined by four days per decade (Burakowski et al., 2008; Hamburg et al., 2013).

The soils at Hubbard Brook are acidic (pH 3.9 to 4.5) with an organic layer consisting of leaf-litter (O_i_), a dense root-mat and decomposing organic material (O_e_), and a nutrient rich humus layer (O_a_) that extends 5 to 6.5 cm below the soil surface (Bohlen et al., 2001). The forest type at Hubbard Brook is northern hardwood forest, with dominant tree species including American beech (*Fagus grandifolia*), sugar maple (*Acer saccharum*), eastern hemlock (*Tsuga canadensis*) and yellow birch (*Betula alleghaniensis*). Red spruce (*Picea rubens*) and balsam fir (*Abies balsamea*) are also found above 740 m.

### Winter Climate Gradient at Hubbard Brook

In this study we used six sites at Hubbard Brook that naturally varied in snowpack depth and duration, soil frost, and soil temperature during winter due to differences in elevation and aspect. The experimental design and site description have been described in detail previously (Durán et al., 2014, 2017; Fuss et al., 2016; Sorensen et al., 2016) and we only provide a brief description here. Three sites were on north-facing slopes at higher elevation (535, 555, and 595 m) and three sites were on south-facing slopes at lower elevation (375, 411, and 511 m). Because plant community type is a well-known driver of soil bacterial and fungal community structure and function (Classen et al., 2015), each site was located in forest stands dominated by *Acer saccharum* (sugar maple) which maximized sugar maple basal area (25–33.2 m^2^ ha^-1^) and minimized the basal area of other common tree species at Hubbard Brook (e.g., yellow birch 0.2–22.9 m^2^ ha^-1^, American beech 0.2–11.4 m^2^ ha^-1^, Schwarz et al., 2003). These sites also had similar soil characteristics (Durán et al., 2014; Sorensen et al., 2016). Sugar maple stands were chosen because sugar maple is an abundant tree species in northern hardwood forests, is sensitive to winter climate conditions, and has significant effects on soil N cycling due to its leaf litter chemistry (Lovett and Mitchell, 2004; Auclair et al., 2010).

Starting in December 2010 and continuing through winter 2013, snow depth was measured using a Federal Snow Sampling Tube (Rickly Hydrological Company, Columbus, OH, United States) at bi-weekly intervals during winter. Soil frost depth was measured using methylene blue dye-filled frost tubes (Rickard and Brown, 1972). Snow depth and soil frost depth data are publically available online http://hubbardbrook.org/data/dataset.php?id~=~136. Soil volumetric water content and soil temperature at 5-10 cm depth were also measured continuously from December 2010 to November 2013 using Decagon 5TM combination probes and Decagon EM50 data-loggers.

### Root Exclusion and Root Ingrowth Cores

Three 1 m × 2 m plots, separated by at least 50 m, were established at all six sites (6 sites × 3 plots per site = 18 plots total) in May 2011. Root exclusion and root ingrowth cores were incubated in all 18 plots (Sorensen et al., 2016). The root exclusion cores (5 cm diameter × 15 cm length) were constructed of nylon mesh (50 μm pore size) that excluded roots from growing into the soil core, but did not prevent hyphal ingrowth. The root ingrowth cores (5 cm diameter × 15 cm length) were constructed of nylon mesh (2 mm pore size) that allowed fine-roots to grow into the soil core. We did not use soil collected from one common location to fill the root cores. Rather, the cores were filled with soil that was collected from near each site and was hand-sorted in the field to remove roots, gravel, and coarse woody debris. The bottom 10 cm of each nylon core was filled with homogenized mineral soil (B-horizon), while the top 5 cm was packed with homogenized organic horizon soil (Oe/A) and packed to target field bulk density (Morse et al., 2015). Cores of each type (root exclusion and ingrowth) were installed in duplicate at all 18 plots along the gradient (6 sites × 3 replicate plots per site x 2 core types per plot x 2 duplicate cores per plot = 72 cores total). We harvested the cores in October 2013, 29 months after installation, in order to minimize the disturbance effect associated with the root core installation and to allow ample time for roots to colonize the root ingrowth cores (*n* = 36 exclusion and 36 ingrowth cores).

### Soil Core Processing and Measurements of Root Biomass, Root Production, and N Transformation Rates

Organic horizon soil (0–5 cm below soil surface, see definition above) and mineral horizon soil (5–15 cm below soil surface) were removed from each core and kept separate during lab processing. A subsample of each soil type from each core was immediately stored at -80°C for DNA extraction and subsequent microbial community analyses. Fine roots ( < 2 mm) were removed using forceps and root biomass (mg dry mass cm^-3^) was determined after drying for 48 h at 60°C. A subsample of each core was also dried at 60°C for 48 h to determine gravimetric water content. Potential nitrification and net N mineralization rates were determined on subsamples of these same soils in short-term lab incubations as described previously (Sorensen et al., 2016). Briefly, 5 grams of soil were incubated over 14 days and then extracted in 30 mL 2 M potassium chloride. Potential net N mineralization and net nitrification rates were determined as the difference in the sum of NH_4_^+^ plus NO_3_^-^ per unit dry soil (mineralization) or NO_3_^-^ per unit dry soil (nitrification) between Day 1 and 14 and divided by the length of the incubation (Sorensen et al., 2016).

### Illumina Library Preparation, Sequencing, and Bioinformatics

Total soil DNA was extracted from three replicate subsamples of each soil sample (3 extractions x 144 soils total) using the Powersoil DNA Extraction Kit (MoBio, Carlsbad, CA, United States) following the manufacturer’s instructions. Replicate soil DNA extracts were combined prior to PCR amplification for each soil core. We amplified both the hyper-variable V4 region of the 16S rRNA gene and the internal transcribed spacer region (ITS1) for identification of bacteria and archaea or fungi, respectively. Modified forward and reverse PCR primers for bacteria and archaea (518F-807) or (ITS1F-ITS2R) for fungi included the forward and reverse Illumina Nextera adapters. PCR amplification conditions were described in detail previously (Smith and Peay, 2014). PCR products were visualized on agarose by staining the amplicons with SYBR green and purified using Solid-Phase Reversible Immobilization (SPRI) paramagnetic beads (DeAngelis et al., 1995; Fisher et al., 2011). Purified PCR products were quantified using the Qubit hs-DS-DNA kit (Invitrogen, Carlsbad, CA, United States) and pooled in equimolar concentrations to create one 16S rDNA bacterial and one ITS rDNA sequencing library. The 16S rDNA and ITS rDNA libraries were sequenced together on a single lane for 250 bp paired-end Illumina Miseq sequencing completed at the Tufts University Genomics Core Facility (Boston, MA, United States).

Reads were trimmed at both the 5’ and 3’ end to the distal sequence priming site using CutAdapt v1.9.1 (Martin, 2012). Additional low quality regions, defined as base quality encoding below PHRED-33, were also removed from each end of the sequence (up to a maximum of 20 bp) using Trimmomatic v0.35 (Bolger et al., 2014). Reads were paired using USEARCH v7.0 (Edgar, 2010). Paired 16S reads (total = 4.5 million paired reads) averaged 250 bp and paired ITS reads (total = 2.7 million paired reads) averaged 226 bp. We used USEARCH to cluster operational taxonomic units (i.e., OTUs) at 97% similarity and to detect and filter out chimeras and singletons. 16S reads clustered into 6345 OTUs (including both bacteria and archaea) and ITS reads clustered into 1479 fungal OTUs. Taxonomy was assigned to OTUs using the assign_taxonomy.py command in QIIME v1.9.0 (Caporaso et al., 2010b) by referencing the Greengenes database (release 13_5) for bacterial and archaea OTU taxonomy assignment or the UNITE database (v7.0) for fungal OTU taxonomy assignment (Koljalg et al., 2005; DeSantis et al., 2006). For 16S sequences, multiple sequence alignment was performed using the PyNAST algorithm and a phylogenetic tree was generated to calculate Faith’s phylogenetic diversity (Caporaso et al., 2010a). Because sampling depth influences abundance based metrics of α-diversity (Gotelli and Colwell, 2001), we rarefied reads to even sampling depth prior to calculating bacterial OTU richness and phylogenetic diversity. Sequencing depth is also known to affect abundance-based measures of β-diversity (McMurdie and Holmes, 2014), so we normalized OTU tables using a cumulative sum scaling method prior to calculating Bray-Curtis distance matrices, which were used used in β-diversity analyses (e.g., permANOVA, db-RDA see below; Paulson et al., 2013). Fungal OTUs were also assigned to functional guilds by comparing fungal OTU taxonomic assignments to annotated databases using FUNguild v1.0 (Nguyen et al., 2016). Functional guilds were assigned to 497 OTUs out of 1299 fungal operational taxonomic units (OTUs) in the organic soil horizon and to 607 out of 1479 OTUs in the mineral soil horizon.

### Statistical Analysis

All statistical analyses were conducted using R v. 3.2.0 (R Core Team, 2010). Bacterial and fungal community composition responses to the effect of elevation, root ingrowth, and their interaction were determined by permutational analysis of variance (permANOVA, permutations *n* = 999) using the using the R package ‘vegan’ (Oksanen et al., 2011). Permutations were restricted by plot as a random-effect in each model. The variance explained by the permANOVA provided a benchmark for variance that could be explained by the categorical variables elevation, root core type, or their interaction. We sought to also test, partition, and attribute the elevation or core type effects among continuous winter climate variables (snow or soil frost), soil microclimate (soil temperature or moisture), or standing root biomass by utilizng distance-based redundancy analysis (db-RDA, permutations *n* = 999). Snow depth and duration or frost depth and duration were converted into a continuous single variable by calculating the integral of the relationship between time (days) and snow (cm) using the R package ‘pracma’ (Borchers, 2011) prior to running the model. Because some predictors were correlated, the db-RDA were performed as univariate models and adjusted R2 was calculated against a null intercept model.

Bacterial or fungal alpha-diversity and abundance were analyzed with a similar rationale to community composition; first, we explored relationships to elevation, root core type, and their interaction and then determined correlations with soil microclimate or root biomass. Bacterial or fungal richness or phylogenetic diversity responses to effects of snow depth and root ingrowth were determined using linear-mixed effects models with plots as the random effect in the model using the R package ‘nlme’ (Pinheiro et al., 2016). Marginal R^2^ values were calculated as the amount of variation accounted for by the fixed-effects either root core type or snow depth and duration divided by the total model variance (Nakagawa and Schielzeth, 2013). Correlations between microbial functional groups and N cycle process rates were similarly determined using linear mixed-effects models with plot as the random effect in the model. The effect of root ingrowth and snow depth on the relative abundance of bacterial and fungal taxonomic orders or fungal functional groups were determined using generalized linear models with a binomial error distribution and log-link function.

## Results

### Winter Snow and Soil Frost Variation

Winter snow depth was negatively related to soil frost depth in the organic horizon (*R*^2^ = 0.86, *P* ≤ 0.001; Supplementary Figure 1a). Frost depth in the mineral soil horizon ( > 5 cm below the surface) declined exponentially as snow depth increased from low to high elevation sites (Supplementary Figure 1b). Average monthly volumetric water content ranged across elevation from approximately 0.25 m^3^ H_2_O m^-3^ soil to 0.52 m^3^ H_2_O m^-3^ soil (Supplementary Figure 1c). Average monthly soil temperature differed across elevation during the early part of the growing season (i.e., April to May), while differences in soil temperature among sites were subtler from May through September (Supplementary Figure 1d). Soil physical and chemical properties (e.g., bulk density, soil and root C:N) did not differ among root ingrowth and exclusion cores or elevation at the time the root cores were collected for microbial community characterization (Table 1 and Supplemental Table 2).

**Table 1:** Physical and chemical soil characteristics of root exclusion and root ingrowth cores.

		Root Exclusion		Root Ingrowth	
*Soil Type*	*Response Variable*	*Median*	*IQR*	*Median*	*IQR*
Organic	Bulk Density	0.13	0.1–0.2	0.14	0.1-0.2
	Soil C:N Ratio	18.9	17.4–19.9	18.6	17.3-20.3
	Root C:N Ratio	24.8	21.9–27.5	24.1	22.3-26.4
	Gravimetric Water	1.9	1.4–2.3	1.3	0.6-1.9
Mineral	Bulk Density	0.22	0.2–0.3	0.24	0.2-0.3
	Soil C:N Ratio	18.4	14.1–20.2	17.70	14.9–20.8
	Root C:N Ratio	31.5	28.5-35.3	25.70	23.5-27.8
	Gravimetric Water	0.6	0.4-0.7	0.5	0.2-0.6

*(Units - bulk density = g dry soil cm^-*3*^, gravimetric water = g H_*2*_O g soil^-*1*^, IQR – interquartile range)*.

### Effect of Root Ingrowth and WinterSnowpack on Bacterial and FungalComposition and Diversity

**Figure 1:**
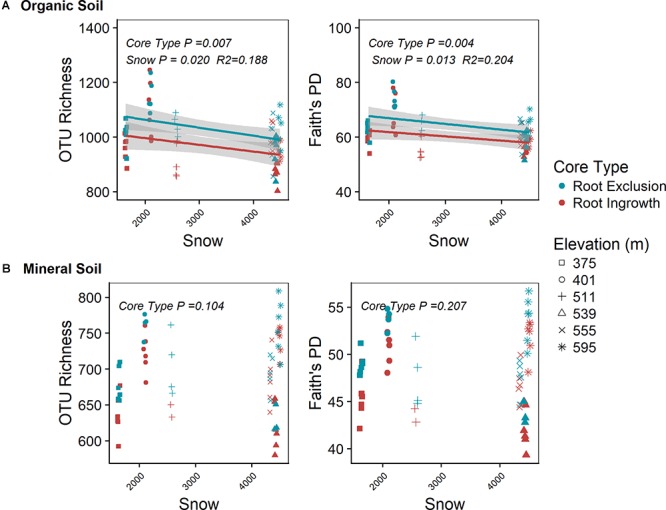
Effect of root ingrowth and snow depth variation on bacterial community diversity in organic **(A)** and mineral **(B)** soil. Points are diversity metrics estimated in individual soil samples and the trend line indicates the linear trend with 95% confidence interval band.

Root exclusion was associated with increases in bacterial richness (Core Type *P* = 0.007) and phylogenetic diversity (Core Type *P* = 0.004) in the organic horizon (Figure 1A). Bacterial richness and phylogenetic diversity also increased with lower snow depth across the elevation gradient in the organic horizon. In the mineral soil horizon, root exclusion likewise led to increases in bacterial richness and phylogenetic diversity, but there was no relationship between snow depth and bacterial richness or phylogenetic diversity (Figure 1B). Elevation was significantly correlated with bacterial community structure in the organic (R2 = 0.42) and mineral soil horizons (R^2^ = 0.50, Supplemental Table 1, Supplementary Figures 2a,b). Snow depth and soil frost explained 14 or 17% (i.e., distance based redundancy adjusted *R*^2^; Table 2, Supplementary Figures 2a,b) of the variation in bacterial composition in the organic horizon and 8% and 14% of the variation in bacterial community composition in the mineral soil horizon.

**Table 2:** Adjusted *R*^2^ determined by distance-based redundancy analysis (db-RDA, permutation *n* = 999).

	Bacterial Organic	Bacterial Mineral	Fungal Organic	Fungal Mineral
*Predictor*	*Adjusted R^2^*	*Adjusted R^2^*	*Adjusted R^2^*	*Adjusted R^2^*
Snow AUC	0.14**	0.08**	0.14**	0.11**
Frost AUC	0.18**	0.14**	0.14**	0.00
Root Biomass	0.00	0.00	0.39**	0.40**
Soil Temperature	0.06**	0.08**	0.11**	0.10
Volumetric Water	0.17**	0.18**	0.01	0.01
Gravimetric Water	0.04**	0.18**	0.12**	0.03

*Response variable was a dissimilarity matrix for bacterial or fungal communities sampled from organic or mineral soil horizons along the elevation and natural winter climate gradient. ^∗^*P* < 0.05, ^∗∗^*P* < 0.01, ^∗∗∗^*P* < 0.001*.

**Figure 2:**
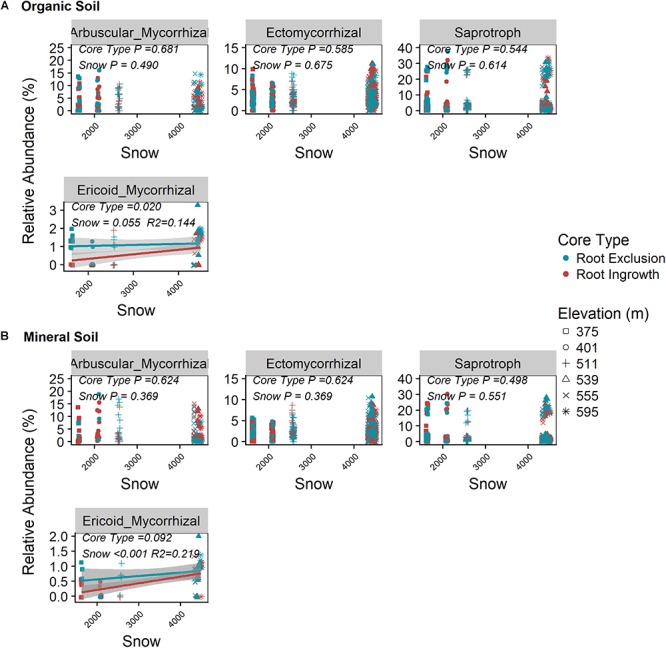
Relationship between snow depth variation and fungal guild abundance in root ingrowth and root exclusion cores in organic **(A)** and mineral **(B)** soil. Fungal OTUs were assigned to guilds using the annotated FUNGuild pipeline. Points are relative abundance measured in soil samples with the linear trend depicted with 95% confidence interval band.

Root exclusion did not have a significant effect on the relative abundance of saprotrophic, arbuscular mycorrhizal, or ectomyccorhizal fungi in either the organic or mineral soil horizons (Figure 2A,B *P* > 0.05, Supplementary Figure S3). By contrast, the relative abundance of ericoid mycorrhizal fungi was greater in root exclusion compared to root ingrowth cores in the organic horizon (Figure 2A) and decreased with decreasing snow depth in both organic and mineral soil horizons (Figure 2A,B).

### Root Influence on the Abundance of Select Bacterial and Fungal Lineages

**Figure 3:**
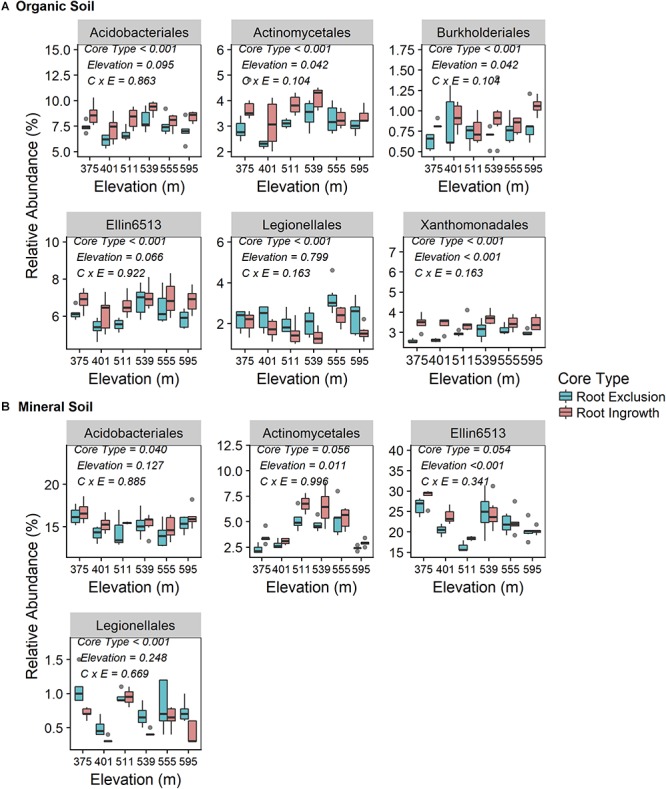
Relative abundance of common (i.e., relative abundance > 0.05%) bacterial taxonomic orders that were significantly affected by root ingrowth in organic **(A)** or mineral soil horizon **(B)**. The boxes span the interquartile range with median depicted by the line in center of the box.

Root exclusion led to consistently lower relative abundance of bacteria in the orders Acidobacteriales, Actinomycetales, and Ellin6513 in both the organic (Figure 3A) and mineral soil horizons (Figure 3B). Similarly, Burkholderiales and Xanthomonadales relative abundance was lower in root exclusion compared to root ingrowth cores, but only in the organic horizon (Figure 3A). By contrast, relative abundance of bacteria in the order Legionellales increased in root exclusion compared to root ingrowth cores across elevation in both organic and mineral soil horizons (Figure 3A,B). The relative abundance of all common bacterial orders (i.e., mean relative abundance > 0.05%) is shown in Supplementary Figure S4.

**Figure 4:**
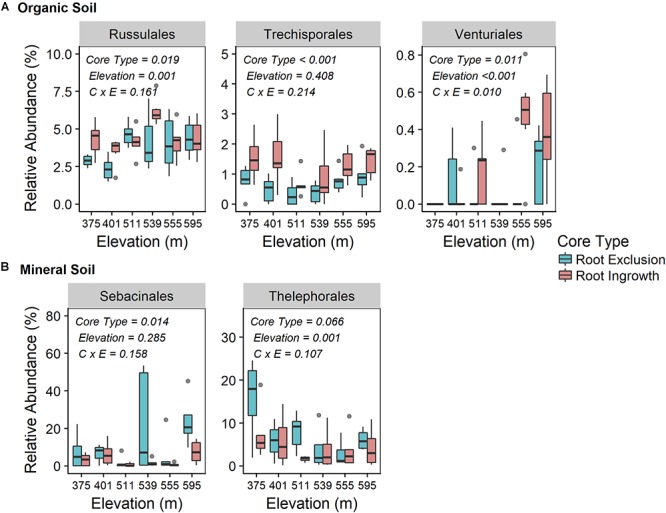
Relative abundance of common (i.e., relative abundance > 0.05%) fungal taxonomic orders that were significantly affected by root ingrowth in organic **(A)** or mineral soil horizon **(B)**. The boxes span the interquartile range with median depicted by the line in center of the box.

Fungal community responses to root exclusion and snowpack variation were subtle. The relative abundance of only three fungal orders (Russulales, Trechisporales, and Venturiales) were affected by root exclusion in the organic horizon (Figure 4A) and two fungal orders (Sebacinales and Thelephorales) were affected by root ingrowth in the mineral soil horizon (Figure 4B). Similar to the bacterial community, elevation was a significant correlate of fungal community structure in both soil horizons (organic R^2^ = 0.24, mineral R^2^ = 0.35, Supplemental Table 1 and Supplementary Figures 2c,d). In contrast to the bacterial community, standing root biomass accounted for a significant amount of the variation in fungal community composition in both organic (adjusted *R*^2^ = 0.39) and mineral soil horizons (adjusted *R*^2^ = 0.40) whereas winter snow and soil frost were less important drivers of fungal community composition compared to standing root biomass (Table 2).

### Winter Snowpack Effects on Bacterial and Fungal Lineages

**Figure 5:**
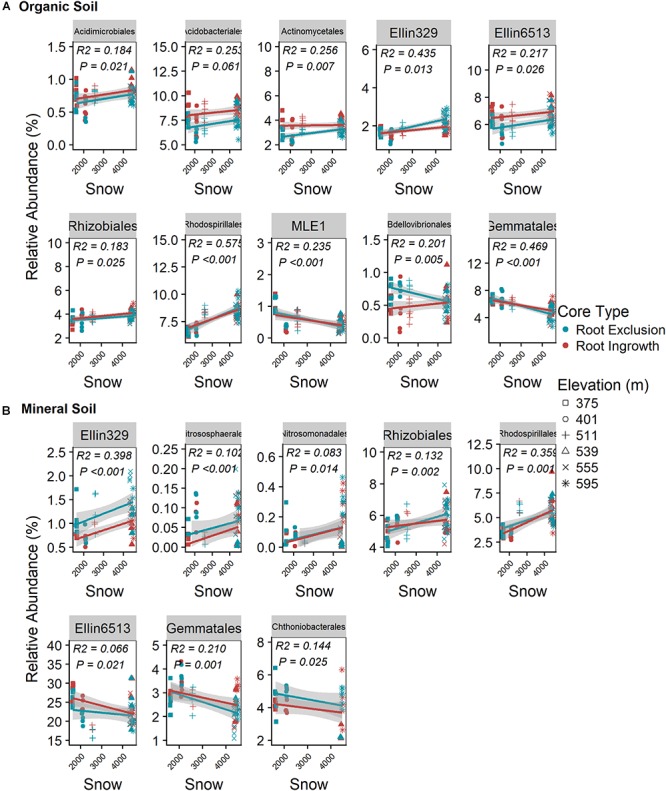
Correlation between snow depth variation and common (i.e., relative abundance > 0.05%) bacterial taxonomic orders in organic **(A)** and mineral **(B)** soil. Points are relative abundance measured in soil samples with the linear trend depicted with 95% confidence interval band.

Snow depth and duration were positively related to the relative abundance of seven bacterial orders in the organic horizon (Figure 5A) and five bacterial orders in the mineral soil horizon (Figure 5B). The positive relationship between relative abundance and snow depth was observed in both organic and mineral soil horizons for the bacterial orders Ellin329, Rhizobiales, and Rhodospirialles. In addition, a significant interaction between root ingrowth and snow depth was observed for Bdellovibrionales abundance in the organic horizon (Figure 5A). Gemmatales abundance increased with declining snow depth in both organic and mineral soil horizons (Figure 5B). Ammonia-oxidizing archaea belonging to the order Nitrososphaerales and bacterial members of the Nitrosomonadales declined with decreasing snow depth in the mineral soil horizons (Figure 5B).

**Figure 6:**
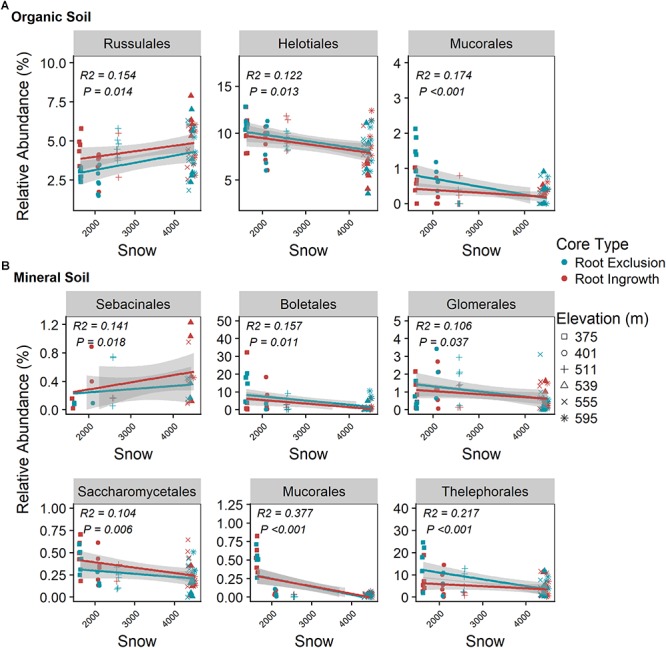
Correlation between snow depth variation and dominate (i.e., relative abundance > 0.05%) fungal taxonomic orders in organic **(A)** and mineral **(B)** soil. Points are relative abundance measured in soil samples with the linear trend depicted with 95% confidence interval band.

Snowpack depth and duration were positively related to Russalales abundance in the organic horizon (Figure 6A) and to the relative abundance of Sebacinales in the mineral soil horizon (Figure 6B). The relative abundance of members of the Helotiales and Mucorales increased with decreasing winter snow in the organic horizon (Figure 6A) as did the relative abundance of Boletales, Glomerales, Sacchormycetales, Mucorales, and Thelephorales in the mineral soil horizon (Figure 6B). The relative abundance of all common fungal genera with associated guild assignment (i.e., mean relative abundance > 0.05%) is shown in Supplementary Figure S5.

### Microbial Functional Groups and Potential Soil N Cycling Rates Across the Elevation Gradient

**Figure 7:**
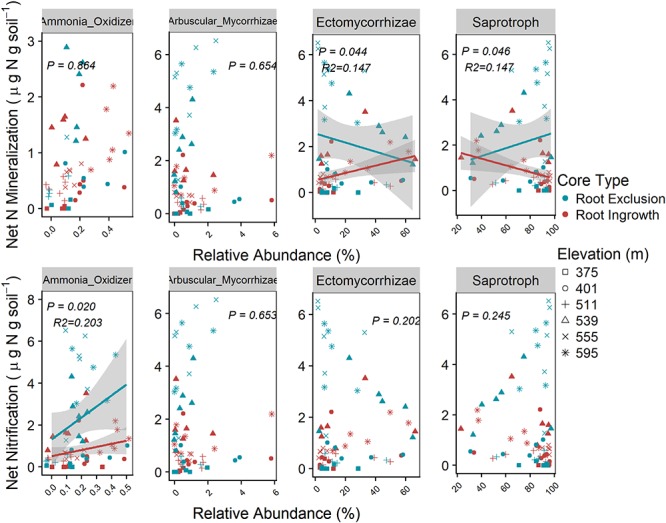
Relationships between potential soil N cycling rates and the relative abundance of microbial functional groups in the organic horizon only. Known bacterial and archaeal ammonia-oxidizing orders (bacteria Nitrosomonades and Nitrospirales and archaea Nitrosphaerales) relative abundance were summed together as Ammonia-Oxidizer abundance. Points are potential N cycle rates and relative abundance measured in soil samples and the line indicates the linear trend with 95% confidence band.

Microbial functional group abundance was related to potential soil N cycling rates in the organic soil horizon only (Figure 7). Root ingrowth interacted with the relative abundance of ectomycorrhizae to increase net N mineralization rates, which was greater at high elevation with more snow compared to low elevation sites with less snow (Figure 7). Conversely, low net N mineralization rates at sites with less snow was inversely related to saprotroph abundance. Potential net N nitrification rates were positively related to the relative abundance of ammonia-oxidizing taxa (e.g., Nitrospirales, Nitrosomonadales, Nitrosphaerales), was higher in root exclusion compared to root ingrowth cores, and decreased with declining depth and duration of the winter snow cover.

## Discussion

A decline in winter snowpack depth and duration has increased the incidence of winter soil freezing in many mid-latitude ecosystems (Henry, 2008; Brown and DeGaetano, 2011; Kreyling and Henry, 2011). In addition, winter soil frost damage to tree roots is known to increase dissolved N in soil solution (Fitzhugh et al., 2001; Tierney et al., 2001; Campbell et al., 2014; Sanders-DeMott et al., 2018). In this study, we found that both root exclusion and a smaller winter snowpack increased bacterial richness and phylogenetic diversity and ericoid mycorrhizal abundance, but the relative abundance of saprotrophic fungi, arbuscular mycorrhizae, or ectomycorrhizal fungi was not related to root exclusion nor winter snow. In addition, root exclusion had a direct negative effect on the relative abundance of several bacterial taxonomic orders, which was typically consistent across elevation zones and in different soil horizons. Although root exclusion did have some effects on fungal taxonomic relative abundance, the effect was less consistent for fungi compared to bacteria and was not consistent across soil horizons. Variation in bacterial community composition was best explained by elevation and a relationship with winter snow depth. By contrast, fungal community composition variation among sites was best explained by standing root biomass. We observed that potential soil N mineralization rates were related to saprotrophic and ectomycorrhizal abundance, while soil nitrification rates were related to the abundance of ammonia-oxidizing bacteria and archaea. Altogether, the results from this study suggest that a declining winter snowpack and its effect on plant roots each impact the diversity and abundance of soil bacteria and fungi that can interact to determine rates of soil N cycling in northern forest ecosystems.

Annual aboveground production (ANPP) at our mid-elevation site (511 m) is higher (716–770 g m^-2^ yr^-1^) compared to the lower elevation and higher elevation sites used in this study (609–662 g m^-2^ yr^-1^, Fahey et al., 2005). Thus, the bacterial and fungal community responses to elevation that we observed are unlikely to be due to differences in ANPP across the sites used in our study. Moreover, snow depth and duration and soil N cycle rates generally increase linearly and positively from low to high elevation sites (Bohlen et al., 2001; Durán et al., 2014; Sorensen et al., 2016). In addition, sugar maple is a tree species known to have significant effects on soil N cycling due to its leaf litter chemistry (Lovett and Mitchell, 2004). We controlled for this important tree species effect by maximizing sugar maple basal area and minimizing the basal area of other common species across our six sites (Schwarz et al., 2003). It is possible that other factors that co-vary with elevation (e.g., % soil organic matter, root production) or aspect (e.g., growing season length) may account for some of the variation in microbial community responses that could not be attributed to elevation or winter climate in our analysis (Richardson et al., 2006; Morse et al., 2015; Sorensen et al., 2016).

Root exclusion led to a few unexpected trends in bacterial and fungal diversity. The presence of roots decreased bacterial richness and phylogenetic diversity, while root exclusion had few observable effects on the relative abundance of fungal functional guilds. One explanation for higher bacterial diversity in soils without roots is that there was a trend for gravimetric water content to be higher in the root exclusion compared to the root ingrowth cores in the organic horizon (Table 1) and increased water availability in soil is known to increase bacterial community diversity (Cruz-Martinez et al., 2009; Castro et al., 2010; Sorensen et al., 2013). Consequently, lower bacterial diversity in the root ingrowth cores may have been in part due to decreased soil moisture as a result of increased root water uptake by plants. In support of this hypothesis, the abundance of some drought-tolerant bacteria (e.g., Actinomycetales, Evans et al., 2014) was consistently greater in root ingrowth compared to root exclusion cores. Proliferation of rhizosphere specialists may have also led to lower bacterial diversity in root ingrowth compared to root exclusion cores.

We observed some root ingrowth and snow depth effects on fungal taxonomic orders that are nominally saprotrophic (e.g., Sacharromycetales, Mucorales) or mycorrhizal (e.g., Russalales, Glomerales, Thelephorales), but the lack of a widespread root ingrowth effect on fungal functional guilds suggests that other traits or trait complexes, in addition to carbon acquisition strategy, mediate fungal functional group distributions at our field site. The seasonal timing of our sampling also has some implications for the inferences we can draw about the impact of snow and soil frost on soil fungal communities. We sampled after plant senescence in October 2013, a period when tree C allocation belowground is likely to be at annual minima (Abramoff and Finzi, 2016), potentially affecting the root-associated fungal communities that we observed. For example, sugar maple associates primarily with arbuscular mycorrhizal fungi and AMF root-colonization, abundance, and biomass are likely to be highest during periods of higher plant C allocation belowground in mid-summer (Mandyam and Jumpponen, 2008; Soudzilovskaia et al., 2015) whereas saprotroph abundance would be expected to increase following root senescence (Fisk et al., 2011).

Ericoid mycorrhizal fungi were a fungal functional guild that showed a consistent and positive association with snow depth, with higher abundance at high elevation sites with more winter snow. The ericoid mycorrhizal community was mainly composed of three fungal genera (Rhyzoscyphus, Sebacinales, and Leptodontidum) that are known to colonize the roots of orchids (Nguyen et al., 2016). Cleavitt et al. (2017) recently observed that two round-leaved orchid species (*Platanthera orbiculate* and *P. macrophylla*) at Hubbard Brook may be experiencing a population bottleneck evidenced by a higher proportion of orchids at adult-compared to juvenile life-stages as well as low juvenile survivorship. Seed establishment and juvenile survivorship of orchids during early mycoheterotrophic life-stages are known to be highly dependent on close association with the fungal symbiont (McCormick et al., 2018), and the results from our study suggest the distribution of these fungal symbionts will decline with future reductions in winter snow depth at Hubbard Brook.

The results from our study provide novel evidence that observed gradients in soil N cycling with elevation in a northern forest are linked to a direct effect of winter climate on tree roots, as well as indirect, root-mediated effects on bacterial N cycle functional groups, such as ammonia-oxidizing bacteria or archaea (e.g., Nitrospirales, Nitrosomonadales, and Nitrosphaerales). Consequently, future reductions in winter snowpack depth can be expected to reduce soil nitrification rates directly by reducing ammonia-oxidizing bacteria or archaea abundance. We also observed significant declines in net N mineralization rates as ectomycorrhizal abundance declined and saprotrophic fungi increased, trends that were driven by reductions in winter snow depth. These patterns may have ecosystem-level implications and consequences for tree N acquisition for species such as sugar maple, which preferentially acquire N in the form of ammonium (Templer and Dawson, 2004). Thus, reduced ammonium supply may limit tree productivity in a future with less winter snow. On the other one hand, increased root production due to winter frost damage may offset a reduction in N supply if increased root production increases root N uptake capacity (Sorensen et al., 2016), and these contrasting outcomes warrant further investigation.

## Conclusion

Because plant roots exert significant influence on both soil bacterial and fungal microbial community composition and structure, incorporating a root-microbial interaction perspective into winter climate change research will lead to a broader understanding of the effect of snowpack decline on northern hardwood forests. Here we provide new evidence that as winter air temperature continues to increase in coming decades, we should expect that plant roots will mediate the response of soil microbial communities to winter snow loss in northern forest ecosystems. Specifically, should winter snow cover continue to decline as projected, our results suggest that the capacity of these soils to mobilize N could be compromised due to the negative effect of soil frost on soil bacteria, archaea and fungi, and that these effects will be driven by both direct effects of snowpack and indirect effects mediated by frost damage to plant roots.

## Author Contributions

PS, AF, and PT conceived of this contribution to the winter climate gradient study at Hubbard Brook that was originally developed and implemented by PG, TF, JD, JM, LC, MF, and PT. The data collection and analysis were performed by PS (primarily), PG, JD, and JM (soil microclimate), and PS and JB (bioinformatics). The manuscript was written primarily by PS and PT and all authors contributed to refining the text.

## Conflict of Interest Statement

The authors declare that the research was conducted in the absence of any commercial or financial relationships that could be construed as a potential conflict of interest.
