# Supplementary material for: Roots Mediate the Effects of Snowpack Decline on Soil Bacteria, Fungi, and Nitrogen Cycling in a Northern Hardwood Forest
Source: Front Microbiol. 2019 Apr 30;10:926. doi: 10.3389/fmicb.2019.00926 (PMC6503048; doi:10.3389/fmicb.2019.00926)
Supplement: Supplementary file 1 [file Data_Sheet_1.zip › Sorensen et al Supplemental R1.docx]

**Supplemental Table 1.** Permutational anova table results of bacterial and fungal community responses to elevation, root core type, and their interaction. Permutations were restricted by ‘Plot’ to account for the hierarchical experimental design.

| Community | Soil Type | Predictor | psuedo-F | R2 |
| --- | --- | --- | --- | --- |
| Bacteria | Organic | Elevation | 9.81 | 0.42 |
|  |  | Core Type | 3.85 | 0.03 |
|  |  | C x E | 0.35 | 0.04 |
|  | Mineral | Elevation | 11.6 | 0.50 |
|  |  | Core Type | 2.1 | 0.02 |
|  |  | C x E | 1 | 0.04 |
| Fungi | Organic | Elevation | 1.21 | 0.24 |
|  |  | Core Type | 3.81 | 0.01 |
|  |  | C x E | 1.02 | 0.06 |
|  | Mineral | Elevation | 1.52 | 0.35 |
|  |  | Core Type | 5.45 | 0.02 |
|  |  | C x E | 1 | 0.06 |

**Supplemental Table 2.** Soil physical and chemical properties measured across elevation.

| Soil Type |  | Bulk Density |  | Soil C:N Ratio |  | Root C:N |  |
| --- | --- | --- | --- | --- | --- | --- | --- |
|  | Elevation | Exclusion | Ingrowth | Exclusion | Ingrowth | Exclusion | Ingrowth |
| Organic | 375 | 0.3 (0.2-0.4) | 0.3 (0.2-0.4) | 17.7 (17.5-18.4) | 18 (16.9-18.2) | 28.0 (24.1-29.0) | 26.0 (24.0-27.7) |
|  | 401 | 0.1 (0-0.2) | 0.1 (0.1-0.2) | 16.7 (16-17.3) | 16.4 (16-17.3) | 20.1 (19.7-20.6) | 21.7 (20.4-22.6) |
|  | 511 | 0.1 (0.1-0.2) | 0.2 (0.1-0.2) | 19.8 (19.5-20.4) | 20.9 (19.5-21.1) | 25.4 (24.8-28.2) | 25.0 (24.0-26.7) |
|  | 539 | 0.1 (0.1-0.2) | 0.1 (0.1-0.2) | 19.5 (18.9-20.3) | 19.8 (19.5-20.2) | 25.1 (22.6-27.6) | 26.5 (24.8-28.7) |
|  | 555 | 0.2 (0.1-0.2) | 0.2 (0.1-0.3) | 20.8 (19.9-21.3) | 20.4 (19.8-20.8) | 23.8 (21.5-26.3) | 23.9 (22.2-24.3) |
|  | 595 | 0.1 (0.1-0.2) | 0.1 (0.1-0.2) | 17.9 (17.2-18.8) | 17.6 (17-18) | 25.0 (22.8-25.4) | 22.7 (22.4-26.3) |
| Mineral | 375 | 0.3 (0.2-0.3) | 0.3 (0.2-0.4) | 17.2 (16.8-18.1) | 17.8 (17.4-18.2) | 39.0 (31.8-39.2) | 28.5 (26.8-31.6) |
|  | 401 | 0.2 (0.1-0.3) | 0.3 (0.2-0.3) | 14.1 (14-15.2) | 14.9 (14.9-15) | 32. 4 (21.4-43.3) | 22.6 (22.4-25.6) |
|  | 511 | 0.2 (0.1-0.3) | 0.2 (0.2-0.3) | 22.3 (21.7-22.4) | 22.2 (21.9-22.9) | 35.4 (32.5-36.3) | 25.2 (24.1-27.7) |
|  | 539 | 0.2 (0.1-0.2) | 0.2 (0.1-0.3) | 20.2 (19.3-20.4) | 19.5 (18.1-21.2) | 31.6 (28.5-32.4) | 27.3 (25.1-28.9) |
|  | 555 | 0.2 (0.2-0.3) | 0.2 (0.2-0.3) | 18.9 (18.7-19.2) | 18.8 (17.2-20.7) | 28.6 (28.6-29.4) | 23.7 (23.5-27.3) |
|  | 595 | 0.4 (0.3-0.4) | 0.3 (0.2-0.4) | 13.1 (12.7-13.4) | 13.1 (12.7-14) | 29.9 (26.1-31.5) | 25.2 (23.9-25.8) |


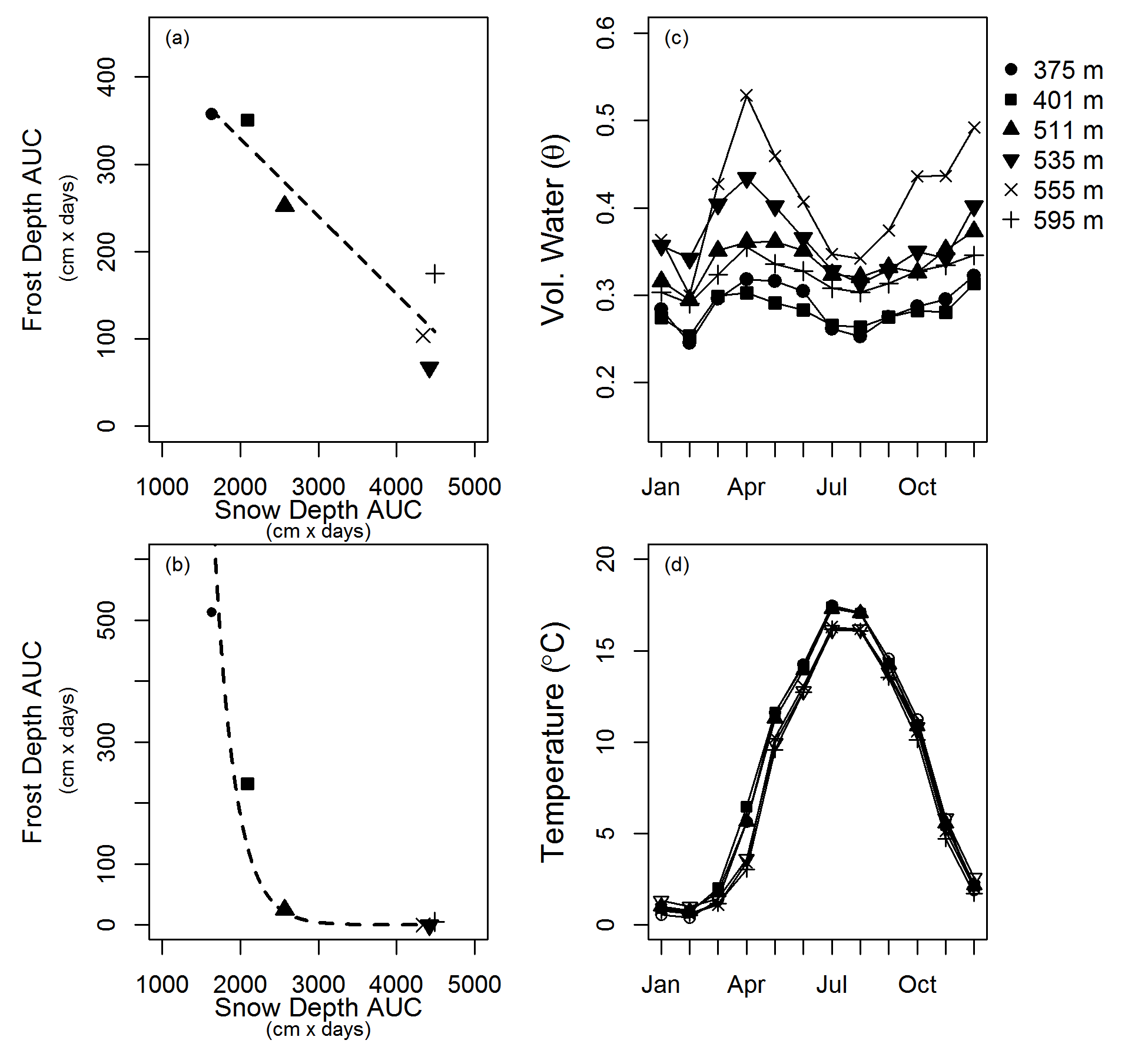
**Supplemental Figure 1 .** Soil microclimate along the winter climate gradient. The area under the curve (AUC) was calculated for snow and soil frost depth measured throughout winter in 2012/13 and the relationship between snow versus frost depth AUC is shown for organic soil (1a) and the mineral soil (1c) separately. Mean monthly volumetric water content (1b) and (1d) soil temperature were calculated from daily mean temperatures measured at site from Dec 2010 to November 2013.


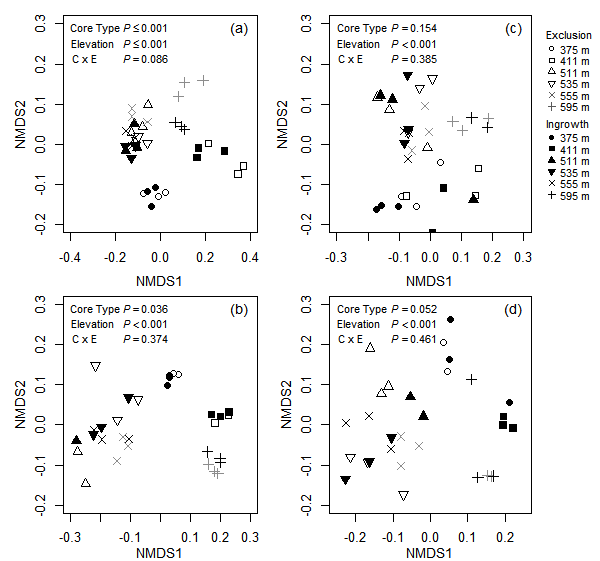
**Supplemental Figure 2** Non metric dimensional scaling for bacterial (3a) or fungal (3c) communities sampled in the organic soil horizon, as well as bacterial (3b) or fungal (3d) communities in mineral soil horizon.

**Supplemental Figure 3** Fungal taxa were assigned to guilds using FUNguild. We assessed the effects of root ingrowth core type and elevation as well as their interactions using a linear mixed effect model, but did not observed significant relationships or interactions among fixed effects. The one exception was ericoid mycorrhizal fungi (see main text).

**
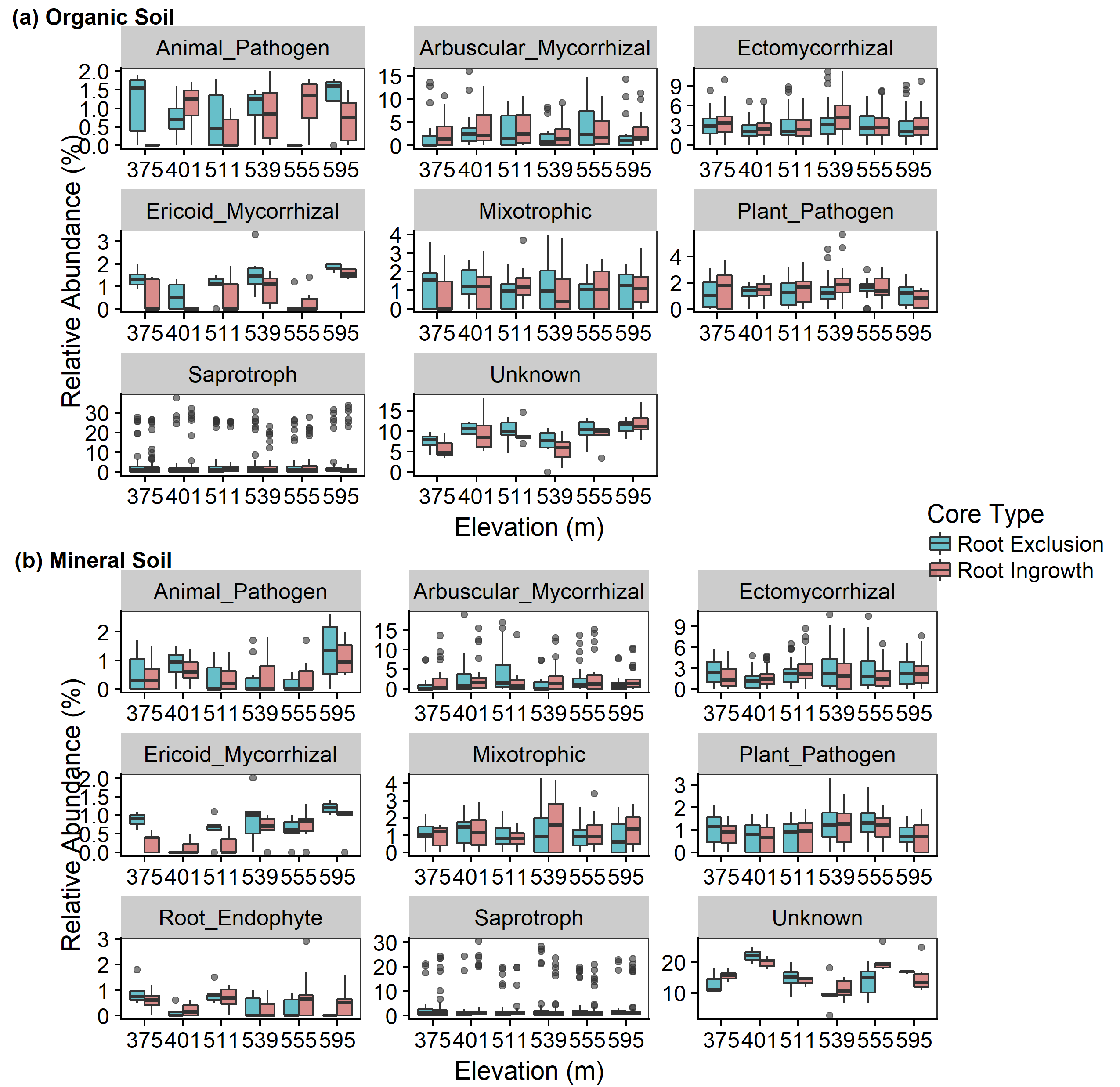
**

**
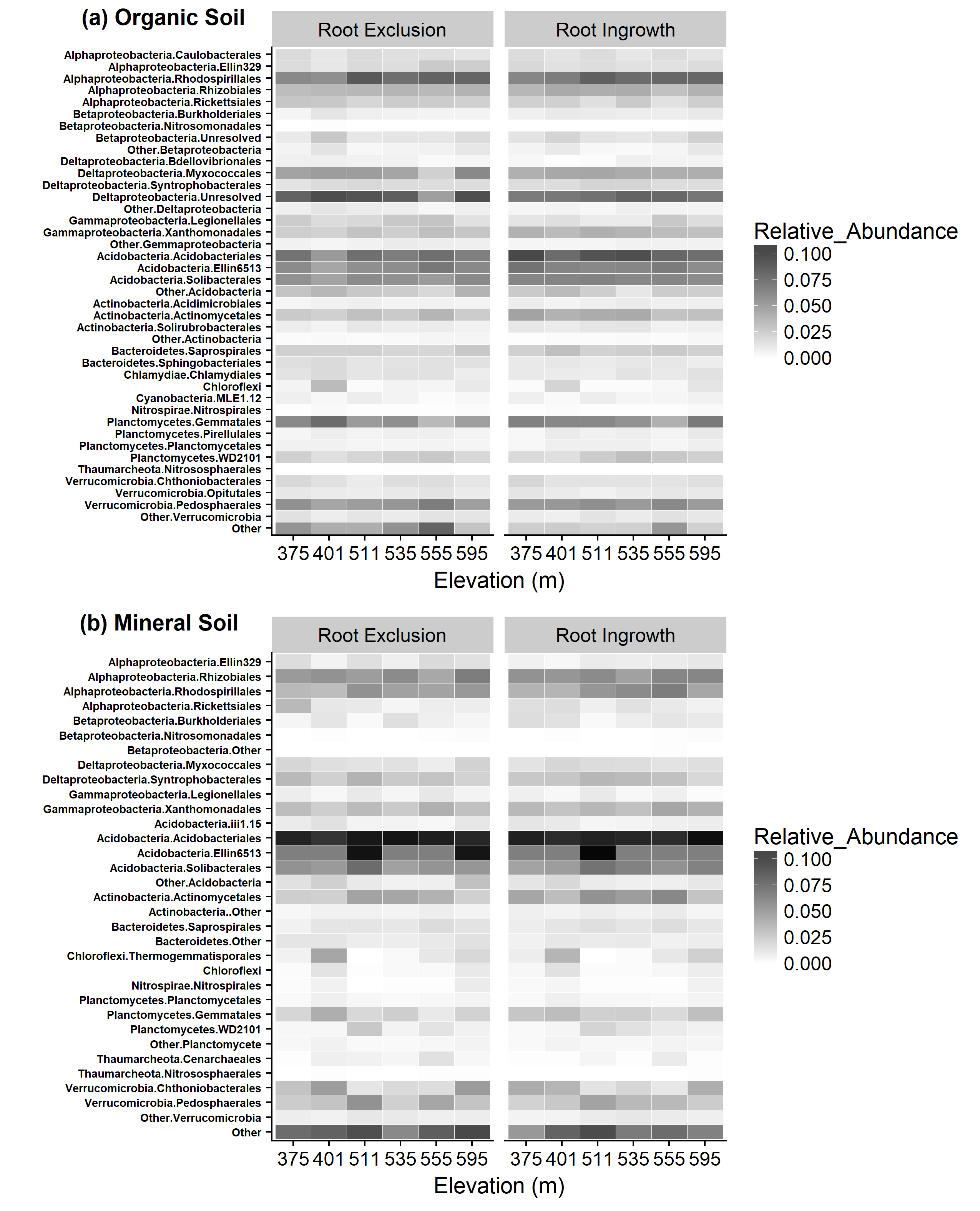
Supplemental Figure 4** Bacterial relative sequence abundance across the winter climate gradient are summarized at the order level of taxonomic resolution. Only bacterial taxa present at ≥ 0.05% relative abundance are summarized here.

**Supplemental Figure 5** Fungal relative sequence abundance across the winter climate gradient were classified into functional guilds (functional guilds precedes genus for each taxa listed on the y-axis). Only fungal taxa present at ≥ 0.05% relative abundance are summarized here.

**
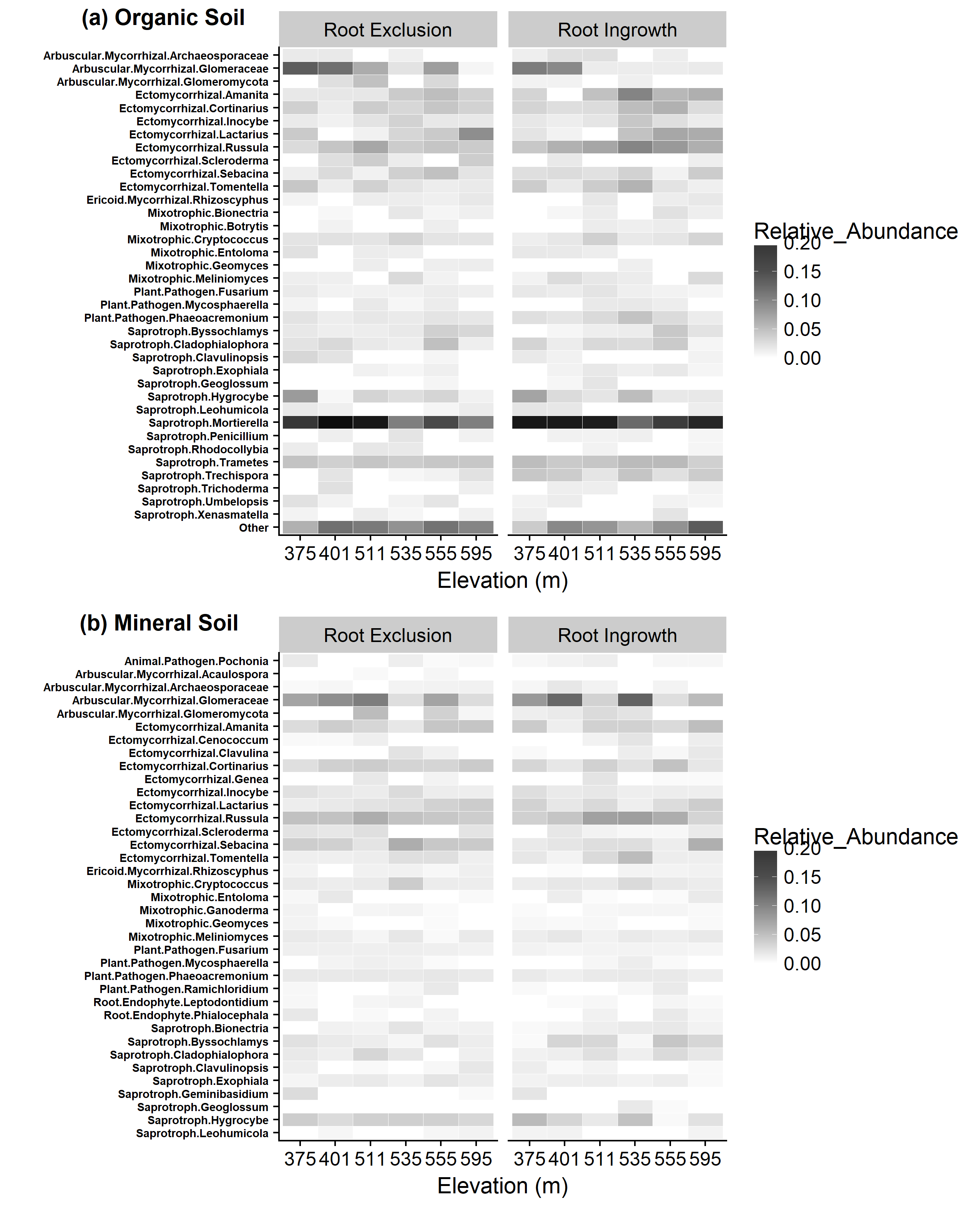
**
